# The Angiogenic Markers PlGF and sFlt-1 in Cytomegalovirus Infection During Pregnancy: Insights from a Clinical Case

**DOI:** 10.3390/v17020267

**Published:** 2025-02-15

**Authors:** Valentina Giardini, Ramona Chiozzi, Federica Fernicola, Marco Casati, Anna Locatelli, Sara Ornaghi

**Affiliations:** 1 Unit of Obstetrics, Foundation IRCCS San Gerardo dei Tintori, 20900 Monza, Italy; 2 Specialization School in Obstetrics and Gynecology, University of Milano-Bicocca, 20900 Monza, Italy; 3 Laboratory Medicine, Foundation IRCCS San Gerardo dei Tintori, 20900 Monza, Italy; 4 School of Medicine and Surgery, University of Milano-Bicocca, 20900 Monza, Italy

**Keywords:** cytomegalovirus, infection, placental dysfunction, angiogenic markers, PlGF, sFlt-1, sFlt-1/PlGF

## Abstract

Cytomegalovirus (CMV) infection during pregnancy is the leading cause of congenital infection subsequent to viral transplacental transmission. CMV placental infection can contribute to the development of adverse outcomes likely through placental dysfunction. This case report shows the potential utility of angiogenic markers, such as placental growth factor (PlGF) and soluble fms-like tyrosine kinase-1 (sFlt-1), in assessing CMV-related placental involvement and monitoring the effect of antiviral therapy on placental function, and highlights the possibility of integrating these markers into the clinical management of CMV infection.

## 1. Introduction

Cytomegalovirus (CMV), a DNA herpesvirus, is the leading cause of congenital infection worldwide, with an estimated overall prevalence of 0.67% [1]. This condition represents a significant global health concern due to its potential to cause severe long-term disabilities in affected neonates, including hearing loss and neurological impairment [2]. Neonatal adverse outcomes in congenital CMV (cCMV) infection are related to the timing of maternal infection during pregnancy. In particular, infections acquired during the periconceptional period and the first trimester are at the highest risk of fetal and neonatal sequelae [3,4].

In the absence of a universal maternal serological screening, suspicion of cCMV infection often arises from fetal ultrasound findings, such as growth restriction, and can be confirmed through amniocentesis [5]. Such diagnosis is crucial for timely intervention with the antiviral drug Valacyclovir, which can improve neonatal outcomes [6].

cCMV occurs when the virus infects and crosses the placenta, thus reaching the fetus. Recent studies have shown that placental infection can indirectly play a role in fetal injury, as CMV infects and replicates in cytotrophoblasts [7] and disrupts their function. This can trigger inflammation, leading to villitis, deciduitis, and ischemia [8]. These conditions cause placental edema and fibrosis, reducing oxygen and nutrient supply to the fetus, thus possibly contributing to the development of fetal growth restriction (FGR) [9].

Given the potential role of placental dysfunction in the pathogenesis of cCMV-related FGR, monitoring angiogenic factors such as placental growth factor (PlGF) and soluble Fms-like tyrosine kinase 1 (sFlt-1) could provide valuable insights into CMV-induced placental pathology.

**Figure 1:**
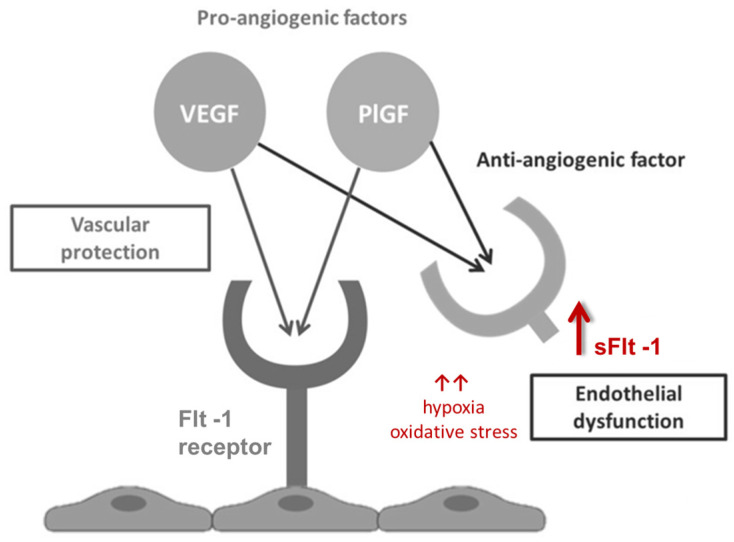
Angiogenic factor interactions.

PlGF is a pro-angiogenic protein from the vascular endothelial growth factor (VEGF) family that supports placental angiogenesis and vascular homeostasis. sFlt-1, a soluble VEGF receptor, antagonizes the positive effects of PlGF, leading to endothelial dysfunction; it is released in response to hypoxia and oxidative stress [10] (Figure 1). The assessment of the sFlt1/PlGF ratio is well-established in the diagnosis and management of the obstetric syndrome of preeclampsia (preE) [11]. Imbalanced angiogenesis is characteristic of normal placental maturation but becomes pathognomonic of placental dysfunction when excessive or premature [12]. Elevated maternal serum levels of sFlt-1 are associated with the onset and severity of preE, while low levels of PlGF are linked to both preE and FGR [13].

This case report explores the potential utility of angiogenic markers, sFlt-1 and PlGF, in assessing placental function in the case of cCMV infection complicated by FGR and undergoing antiviral treatment with Valacyclovir.

## 2. Detailed Case Description

A 19-year-old primigravida with an unremarkable medical and surgical history and spontaneous conception was diagnosed with FGR according to Delphi criteria [14] at 27^6/7^ weeks’ gestation.

The woman had a pre-pregnancy body mass index of 18.1 kg/m^2^ and was a non-smoker. Her partner, non-consanguineous, was in good health. Family history was negative for hereditary diseases or congenital malformations. She had worked as a babysitter for a toddler up to the second trimester.

Pregnancy dating was based on her last menstrual period (regular cycles), with a positive pregnancy test one month after her last period and an ultrasound at 12^5/7^ weeks showing a crown-rump length consistent with 12 weeks. The woman decided not to undergo any non-invasive prenatal diagnosis testing.

At the 20^3/7^ week ultrasound, fetal anatomy was normal; however, fetal biometrics were at the lower limits according to Hadlock’s curves [15,16]: abdominal circumference (AC) was at the 6th centile, biparietal diameter (BPD) and head circumference (HC) were below the 10th centile, and the femur length (FL) was at the 20th centile. Maternal–fetal Doppler velocimetry values were normal. Amniocentesis was not performed, in line with the mother’s will; also, maternal CMV serology was not investigated.

At 27^6/7^ weeks, FGR was diagnosed with an AC and estimated fetal weight (792 g) below the 1st centile. Despite a generally low growth trend, fetal growth continued until 35^4/7^ weeks when a plateau was detected, associated with vasodilation in the middle cerebral artery, prompting hospital admission for more intensive monitoring. The ultrasound showed no additional abnormalities, and maternal Doppler velocimetry values were normal. Maternal blood pressure was regular.

During hospital stay, blood pressure remained normal, and routine laboratory tests showed only mild anemia.

To gain a better understanding of the potential underlying cause of FGR, both angiogenic markers (Cobas e601 platform, Roche Diagnostics—Basel, Switzerland) and maternal CMV serology and virology (PCR, CMV ELITe MGB Kit, ELITechGroup—Puteaux, France) were assessed to investigate the placental and infectious cause, respectively. The sFlt-1/PlGF ratio at 35^6/7^ weeks was 106.18, an intermediate value according to Verlohren’s categories [17] (Table 1). PlGF was 94.8 pg/mL, slightly below the 10th centile for gestational age in an uncomplicated singleton pregnancy, but above the 10th centile compared to a term singleton pregnancy (68.6 pg/mL). In contrast, sFlt-1 levels were 10,006 pg/mL, above the 90th percentile for both gestational age and an uncomplicated singleton term pregnancy (7901 pg/mL) [18] (Table 2). Maternal CMV serology showed positive IgG and IgM (IgG 99.6 U/mL, CLIA, positive ≥ 22; IgM 28 U/mL, CLIA, positive ≥ 14), with IgM positivity confirmed with an alternative test (IgM 0.94 index, ELFA, positive >0.9) and intermediate IgG avidity (0.189 index; low avidity < 0.150, high avidity > 0.250). Viral DNA was identified in urine (1350 UI/mL), whereas blood and saliva were negative. These laboratory findings were suggestive of a maternal infection in pregnancy, which prompted the execution of an amniocentesis at 365/7 weeks to assess the fetal infectious status and its karyotype. The virological assessment of the amniotic fluid revealed CMV DNA at high levels (206,000,000 copies/mL); the molecular karyotype was normal. A detailed ultrasound examination did not identify any additional fetal abnormalities.

Considering the diagnosis of symptomatic cCMV infection (i.e., FGR) and in line with the Italian Drug Agency statement on the use of Valacyclovir in pregnancy [19], the woman was started on antiviral therapy (2 g every 6 h) until childbirth.

At 37^6/7^ weeks’ gestation, 8 days after starting Valacyclovir, angiogenic markers were assessed again, revealing an improvement in the angiogenic profile with both PlGF and sFlt-1 within the range for an uncomplicated singleton term pregnancy (114.0 pg/mL and 7303.0 pg/mL, respectively), and an intermediate sFlt-1/PlGF ratio according to Verlohren’s categories (64.06) [17,18] (Table 3).

The 38^0/7^ week-ultrasound evaluation showed an estimated fetal weight of 1866 g (<1st centile according to Hadlock) [16], and a persistent middle cerebral artery vasodilation. Thus, at 38^2/7^ weeks of gestation, labor was medically induced, leading to a vaginal birth of a female newborn weighting 1900 g (<1st centile according to INTERGROWTH-21 charts [20]), with a regular Apgar score and umbilical artery pH.

The patient’s postpartum course was uncomplicated, and she was discharged on the third day after birth, while the neonate was admitted to the neonatal post-intensive care unit for further evaluation. PCR testing for CMV DNA in neonatal blood, urine, and saliva was positive (blood, 4161 copies/mL; urine, 212,000,000 copies/mL; saliva 20,575,137 copies/mL), confirming cCMV infection. Laboratory results revealed mild, transitory thrombocytopenia. Cerebral ultrasound and MRI were negative, with an unremarkable neurological clinical examination. Similarly, additional exams, including abdominal ultrasound, ophthalmological examination with fundus oculi, and hearing screening with otoacustic emission and auditory brainstem response were normal. Thus, postnatal antiviral treatment was not administered. Follow-up at 24 months was regular, with appropriate neurodevelopment score (Bayley-III) and normal hearing.

Placental histology revealed mild chronic villitis with a stromal infiltrate of CD3-positive lymphocytes and rare CD138-positive plasmacytic cells, along with microclots in villous vessels and some fibrotic, avascular villi.

## 3. Discussion

This case highlights the potential role of angiogenic markers in the context of intrauterine CMV infection. Although these markers provide valuable insights into placental function, their routine clinical use remains inconsistent due to ongoing questions regarding interpretation, cut-off values, and performance in conditions such as infections [21].

High sFlt-1 levels have been observed in maternal infections such as COVID-19, where they are associated with a preE-like syndrome [22]. Additionally, elevated sFlt-1 levels have been documented in cases of maternal infection with viruses that replicate in the placenta [23]. Three case reports described women with mirror syndrome, two of which were associated with CMV infection and one with Parvovirus B19 infection, highlighting the potential connection between viral placental infection and altered angiogenic marker levels. Collectively, these cases suggest that an infected, hydropic placenta can secrete exceptionally high levels of sFlt-1 [24,25,26].

In our case, the initial angiogenic profile was characterized mainly by high levels of sFlt-1 (10,006 pg/mL), which was above the 90th centile for both gestational age and an uncomplicated singleton term pregnancy (7901 pg/mL) [18], alongside with a slightly decreased PlGF value (94.8 pg/mL) for gestational age (<10th but above the 5th centile). This angiogenic profile is not typical of placental-related FGR cases, which are usually characterized by very low levels of PlGF [13].

It is possible that PlGF is not substantially impacted by placental CMV infection, since CMV replicates in the cytotrophoblast whereas PlGF is produced by the syncytiotrophoblast. In addition, placental CMV infection has been shown to directly and indirectly enhance all stages of angiogenesis, including the induction of the release of angiogenic factors like VEGF and IL-6, thus leading to pathological angiogenesis [27]. In turn, virally induced placental abnormalities, like villitis, deciduitis, and ischemia, could induce elevated sFlt-1 levels, as this anti-angiogenic molecule is released in response to hypoxia and oxidative stress [10].

Altogether, these findings suggest that angiogenic markers could be used in cases of FGR to aid in the differential diagnosis and better define the extent of CMV-induced placental damage. Of note, sFlt-1 concentration in maternal blood has been recently suggested as a potential predictive biomarker of transplacental CMV transmission in cases of maternal infection in pregnancy [28]. This study showed significant differences in sFlt-1 levels between CMV-transmitting and non-transmitting women; in addition, the authors reported that the sFlt1/PlGF ratio was not altered by transmission, although some cases of transmission were associated with elevated values >38, thus confirming our observation that PlGF does not undergo substantial changes in the case of placental CMV infection. Our case report emphasizes the importance of analyzing both markers and not just the sFlt-1/PlGF ratio and comparing their values with those of a singleton uncomplicated pregnancy at the same gestational age and at term [29,30].

Assessing sFlt-1 levels enables the identification of patients at high risk for preE. The excessive production of sFlt-1 leads to maternal endothelial injury, which contributes to the development of preE manifestations [31], and serves as a strong predictor of both preE severity and adverse outcomes. There appears to be a threshold beyond which elevated sFlt-1 levels become toxic to the mother. Our research demonstrated that increased sFlt-1 levels (≥15,802 pg/mL) are associated with severe obstetric complications in multiple pregnancies with hypertensive disorders of pregnancy (HDP) and FGR, irrespective of gestational age and chorionicity [32]. These findings emphasize the importance of monitoring sFlt-1 levels to predict potential adverse outcomes.

In our case, the sFlt-1 levels were above the 90th centile for both gestational age and an uncomplicated singleton term pregnancy, but below 15,802 pg/mL, and the patient did not develop preE. Interestingly, several observational and experimental studies have reported that CMV infection can constitute a risk factor for the development of preE, hypertension, and cardiovascular disease [33].

Another interesting aspect of this case report is the effect of the antiviral therapy with Valacyclovir on placental angiogenesis. After only 8 days of treatment, the angiogenic placental markers were already within the range for gestational age, thus suggesting that these markers’ levels can vary over time with appropriate therapies. By reducing sFlt-1 levels, antiviral therapy could reduce placental damage, thus possibly averting the development of preE [34].

## 4. Conclusions

This case report provides new insights into the role of angiogenic markers, PlGF and sFlt-1, in maternal and fetal CMV infections. Specifically, it supports their role in the differential diagnosis of FGR and in providing meaningful insights into the severity of CMV-induced placental damage. It also highlights their potential ability in monitoring the effects of the antiviral therapy on placental function. Additionally, this case report underscores the importance of assessing both angiogenic markers separately, and not only their ratio, and comparing them with values from an uncomplicated pregnancy at the same gestational age and at term.

However, it is important to acknowledge that the findings are based on a single case report, and therefore, further research is needed to explore placental angiogenesis in greater depth. In the meantime, it is crucial to educate obstetricians and neonatologists on the interpretation and clinical application of these markers.

## Figures and Tables

**Table 1:** Current categories for sFlt-1/PlGF ratio with relative cut-offs distinguished by gestational age, < or ≥34th week [17].

Risk Category	sFlt-1/PlGF	
	<34th Week	≥34th Week
Low	<38	<38
Medium	38–85	38–110
High	>85	>110
Very high	>655	>201

**Table 2:** Serum reference range 10th–90th percentile for PlGF (pg/mL) and sFlt-1 (pg/mL) across different weeks of pregnancy (Roche Diagnostics) [18].

Gestational Weeks	PlGF (pg/mL)	sFlt-1 (pg/mL)
	Percentile 10–90	Percentile 10–90
10–14	31.3–100.0	776–2174
15–19	80.9–251.0	844–2453
20–23	143–500.0	718–2605
24–28	200.0–921.0	722–2557
29–33	139.0–1073.0	967–3650
34–36	98.2–831.0	1220–5620
37–delivery	68.6–620.0	1899–7901

**Table 3:** Trends of angiogenic markers sFlt-1, PlGF, and sFlt-1/ PlGF before and after antiviral therapy.

Timing of the Dosage	Weeks	sFlt-1	Range Comparison	PlGF	Range Comparison	sFlt-1/PlGF	Risk Category
		pg/mL	Percentile 10–90	pg/mL	Percentile 10–90		
Before Therapy	35^6/7^	10,006	>	94.8	<	106.18	Medium
After Therapy	37^6/7^	7303	•	114.0	•	64.06	Medium

Legend: range comparison for gestational age [18]—“>”: above range; “<”: below range; “•”: within range.

## Data Availability

The data that support the findings of this study are available on request from the corresponding author.
